# Changes in tumor-to-blood ratio as a prognostic marker for progression-free survival and overall survival in neuroendocrine tumor patients undergoing PRRT

**DOI:** 10.1007/s00259-023-06502-y

**Published:** 2023-11-10

**Authors:** Manuel Weber, Olof Pettersson, Robert Seifert, Benedikt M. Schaarschmidt, Wolfgang P. Fendler, Christoph Rischpler, Harald Lahner, Ken Herrmann, Anders Sundin

**Affiliations:** 1 Department of Nuclear Medicine, University of Duisburg-Essen and German Cancer Consortium (DKTK)-University Hospital Essen, Hufelandstraße 55, 45147 Essen, Germany; 2 Department of Surgical Sciences, Uppsala University, Uppsala, Sweden; 3 Institute of Diagnostic and Interventional Radiology and Neuroradiology, University Hospital Essen, Essen, Germany; 4 Department of Endocrinology and Metabolism, Division of Laboratory Research, University of Duisburg-Essen and German Cancer Consortium (DKTK)-University Hospital Essen, Essen, Germany

**Keywords:** NETs, PRRT, Theranostics, Therapy monitoring, DOTATOC PET

## Abstract

**Background:**

Historically, patient selection for peptide receptor radionuclide therapy (PRRT) has been performed by virtue of somatostatin receptor scintigraphy (SRS). In recent years, somatostatin receptor positron emission tomography (SSTR-PET) has gradually replaced SRS because of its improved diagnostic capacity, creating an unmet need for SSTR-PET-based selection criteria for PRRT. Tumor-to-blood ratio (TBR) measurements have shown high correlation with the net influx rate Ki, reflecting the tumor somatostatin receptor expression, to a higher degree than standardized uptake value (SUV) measurements. TBR may therefore predict treatment response to PRRT. In addition, changes in semiquantitative SSTR-PET parameters have been shown to predate morphological changes, making them a suitable metric for response assessment.

**Methods:**

The institutional database of the Department of Nuclear Medicine (University Hospital Essen) was searched for NET patients undergoing ≥ 2 PRRT cycles with available baseline and follow-up SSTR-PET. Two blinded independent readers reported the occurrence of new lesions quantified tumor uptake of up to nine lesions per patient using SUV and TBR. The association between baseline TBR and changes in uptake/occurrence of new lesions with progression-free survival (PFS) and overall survival (OS) was tested by use of a Cox regression model and log-rank test.

**Results:**

Patients with baseline TBR in the 1st quartile had a shorter PFS (14.4 months) than those in the 3rd (23.7 months; *p* = 0.03) and 4th (24.1 months; *p* = 0.02) quartile. Similarly, these patients had significantly shorter OS (32.5 months) than those with baseline TBR in the 2nd (41.8 months; *p* = 0.03), 3rd (69.2 months; *p* < 0.01), and 4th (42.7 months; *p* = 0.03) quartile. Baseline to follow-up increases in TBR were independently associated with shorter PFS when accounting for prognostic markers, e.g., RECIST response (hazard ratio = 2.91 [95%CI = 1.54–5.50]; *p* = 0.01). This was confirmed with regard to OS (hazard ratio = 1.64 [95%CI = 1.03–2.62]; *p* = 0.04). Changes in SUVmean were not associated with PFS or OS.

**Conclusions:**

Baseline TBR as well as changes in TBR were significantly associated with PFS and OS and may improve patient selection and morphological response assessment. Future trials need to assess the role of TBR for therapy monitoring also during PRRT and prospectively explore TBR as a predictive marker for patient selection.

**Supplementary Information:**

The online version contains supplementary material available at 10.1007/s00259-023-06502-y.

## Introduction

The positive results of the NETTER-1 [1] and ERASMUS trials [2] have led to the approval of peptide receptor radionuclide therapy (PRRT) for gastroenteropancreatic neuroendocrine tumors (NETs). In these studies, sufficient target somatostatin receptor expression as a prerequisite for treatment was assessed with [^111^In]In-DTPA-octreotide scintigraphy (SRS).

However, over the past years, SRS has been gradually replaced by somatostatin receptor positron emission tomography (SSTR-PET) due to its higher spatial resolution and superior sensitivity [3–5]. This leads to considerable discrepancies in the assessment of uptake intensity by SRS vs. SSTR-PET, especially in lesions near the gamma camera detectors and those smaller than 2 cm, [6] making SSTR-PET-based selection criteria much needed.

While some studies have shown that uptake intensity on SSTR-PET, quantified as the standardized uptake value (SUV), is predictive of treatment response [7–9] and progression-free survival (PFS) [7], these findings have not been replicated by others [10–12]. Similarly, in two prior studies, baseline to follow-up changes in SUV failed to predict patient outcome in NET patients treated with PRRT [10, 11]. A possible explanation for these observations is that no correlation between SSTR expression and SUV has been shown at higher SUV, which is particularly relevant in PRRT patients, in whom intense SSTR expression is a prerequisite [13]. In contrast, SSTR expression is highly correlated with the net influx rate, Ki [13]. As the latter has been shown to be linearly related to the tumor-to-blood ratio (TBR), TBR might be superior to SUV to predict patient outcome and monitor the effect of treatment [14].

Follow-up after treatment is routinely carried out with cross-sectional imaging, usually computed tomography (CT) or magnetic resonance imaging (MRI). However, the improved diagnostic performance of SSTR-PET over cross-sectional imaging might also aid in treatment monitoring.

The aim of this retrospective single-center study was to evaluate the predictive value of baseline TBR and of baseline to follow-up changes in TBR, with regard to treatment response, PFS, and overall survival (OS) in NET patients undergoing PRRT.

## Materials and methods

### Patients

The institutional database of the University Clinic Essen was screened for NET patients, who have undergone at least two cycles of [^177^Lu]Lu-DOTA-TOC treatment and DOTATOC-PET/CT for baseline and follow-up imaging. The study was performed in accordance with the principles of the Declaration of Helsinki. The local ethics committee (University of Duisburg-Essen, Medical Faculty; protocol number: 22–10,737-BO) approved the analysis of available patient data; the requirement to obtain informed consent was waived.

### Radiopharmaceutical preparation

In-house synthesis of ^68^ Ga peptides followed the procedure outlined by Zhernosekov et al. [15]. ^68^ Ga was obtained from a ^68^Ge/^68^ Ga radionuclide generator (GalliaPharm), and the peptide was supplied by Bachem (until 2016, thereafter by ABX GmBH). The preparation time took approximately 60 min until 2016 and 20 min after; the radiochemical yield ranged from 60 to 70%. Radiochemical yield was not assessed after 2016, but typically, activities ranging from 600 to 1300 MBq were obtained. Quality control assessments, conducted using two thin-layer chromatography (and additionally high-performance liquid chromatography after 2016) systems, confirmed a radiochemical purity exceeding 98%.

### Image acquisition

[^68^ Ga]Ga-DOTA-TOC-PET/CT was performed after administration of mean (± standard deviation (SD)) 75.1 (± 13.8) MBq [^68^ Ga]Ga-DOTA-TOC (equivalent to 1.0 ± 0.3 MBq/kg_bodyweight_) with a mean (± SD) uptake interval of 56.0 (± 25.5) min. In the majority of patients, all follow-up imaging was performed by the use of [^68^ Ga]Ga-DOTA-TOC-PET/CT as well. Until 2019, patients were encouraged to withhold long-acting somatostatin analogs (SSA) in the 4 weeks leading up to SSTR-PET. With the advent of prospective data showing no detrimental effect of SSA administration shortly before SSTR-PET, this practice was not upheld anymore [16].

### Image analysis

Image analysis was performed by two readers (MW, OP) using OsiriX MD (Pixmeo SARL) and Carestream Picture Archiving and Communication System (PACS) for image analysis. The readers were blinded for all clinical and imaging information except that the Ga-[^68^ Ga]Ga-DOTA-TOC examinations had been performed in NET patients undergoing PRRT.

The PET/CT assessment was performed as follows: Reference organ uptake was assessed by measuring SUVmax and SUVmean in a volume of interest with a 1 cm (left ventricle, kidney, spleen), 2 cm (liver), or adaptive (abdominal aorta) radius; an additional 50% threshold was employed for SUVmean measurements of the abdominal aorta.

Tumor uptake was categorized as high (≥ 2 × kidney uptake), intermediate (≥ kidney uptake, but < 2 × kidney uptake), and low (< kidney uptake, but ≥ liver uptake). SUVmax and SUVmean (using a 50% threshold) were measured on baseline, follow-up scans for up to three lesions of each uptake category, i.e., up to nine lesions per examination.

The ratios between SUVmean of the tumor were divided by the SUVmean of the left ventricle (TBR) and were subsequently calculated for each lesion. Based on the high deviation of abdominal aorta uptake values and a high congruence of left ventricle uptake measurements, among readers in a prior exploratory blinded reading of 30 patients, SUVmean of the left ventricle was chosen for TBR calculation over SUVmean of the abdominal aorta.

Repeat blinded readings were then carried out by both readers for all patients with conflicting results, i.e., increasing TBR reported by one reader and decreasing TBR by the other or description of new lesions by one reader but not the other. The remaining conflicting results were settled in a joint consensus session. Lastly, the average per patient baseline to follow-up change of TBR across all individual lesional TBRs was calculated.

### Outcome parameters

TBR at baseline as well as changes in TBR from baseline to follow-up imaging were correlated with PFS and OS. Herein, OS was defined as the interval from the first treatment cycle until death/last follow-up. PFS was defined as the interval from treatment start until at least one of the following progression criteria was met:

1. Occurrence of progressive disease (PD) according to RECIST 1.1 criteria, as determined by consensus of one unblinded central reader (M.W.) and the clinical reads, each validated by a board-certified radiologist and a board-certified nuclear medicine physician [17]
2. Clinical progression as ruled by an experienced board-certified endocrinologist (HL)
3. Interdisciplinary tumor board decision for treatment shift due to combined clinical and radiological progression, without RECIST 1.1 criteria for PD being met
4. Death

### Treatment protocol

Treatment was performed in accordance with European Association of Nuclear Medicine guidelines on PRRT and more specifically, according to the NETTER-1 treatment protocol starting in 2017. Co-infusion of 25 g lysine and 25 g arginine was used for nephroprotection. In the pre-NETTER-1 era, patients routinely underwent PRRT with two cycles, with additional cycles being performed in cases of insufficient treatment response. In the recent decade, the PRRT protocol has shifted towards increasing use of four cycles of [^177^Lu]Lu-DOTA-TOC.

The 139 patients underwent a total (mean) of 470 (3.4) PRRT cycles. The mean cumulative activity per patient was 23.1 GBq [^177^Lu]Lu-DOTA-TOC.

### Statistical analysis

Statistical analysis was performed using SPSS version 27.0 (IBM Corp. Released 2020; IBM SPSS Statistics for Mac, version 27.0, Armonk, NY; IBM Corp).

The impact of mean TBR at baseline as well as changes in TBR and SUVmean on PFS and OS was tested using a univariate and multivariate Cox regression model. PFS and OS of patients with decreasing vs. stable/increasing TBR from baseline to follow-up imaging were compared by use of log-rank test and plotted using Kaplan–Meier curves. Based on a previously published test–retest study, in which unspecific fluctuations in lesional SUVmax of up to 25% were observed, the category of stable TBR (increase/decrease < 25%) was introduced, and additional log-rank tests carried out [18]. A one-way ANOVA with Bonferroni correction was performed to assess differences in TBR for patients with complete/partial remission, stable disease, and PD on first response assessment after PRRT. Pearson’s correlation coefficient was calculated to assess a potential association between SUVmean of the blood pool on the one hand and time post-injection and body weight on the other hand. A *p* < 0.05 was considered statistically significant.

## Results

### Patient cohort

In total, 139 patients were eligible for evaluation; 78/139 (56%) were male and 61/139 (44%) were female. Mean patient age was 62.1 (27.8–84.4) years. The primary NET was located in the pancreas, midgut, and lungs in 47/139 (34%), 40/139 (29%), and 12/139 (9%) patients, respectively. Histopathological grade was G1, G2, and G3 in 35/139 (25%), 75/139 (54%), and 9/139 (6%) patients, respectively, and unknown/not standard practice in the remainder. The patient selection process is shown in Supplemental Fig. 1. An overview of the patient baseline characteristics is provided in Table 1.

**Fig. 1:**
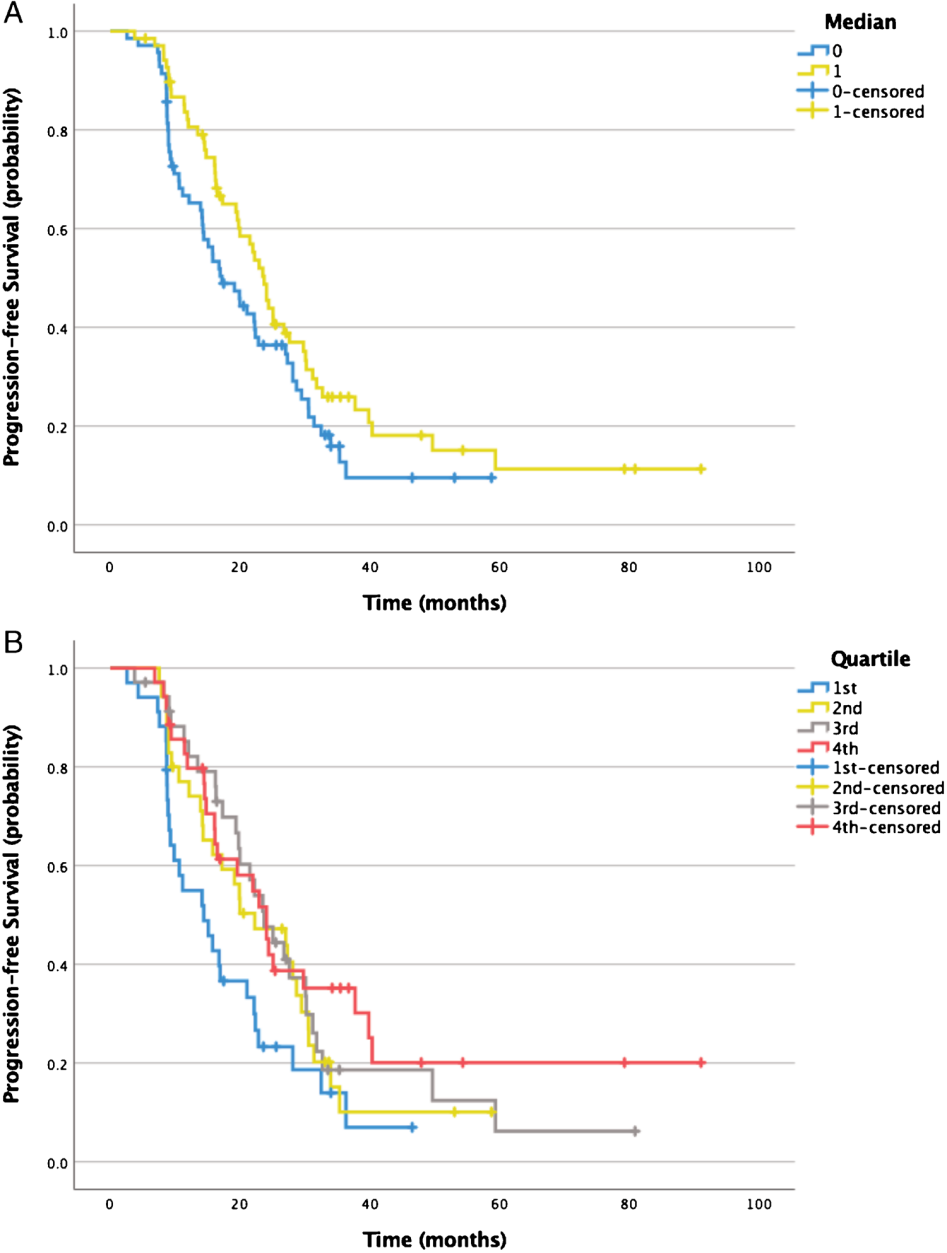
Kaplan–Meier curves showing no statistically significant differences with regard to PFS in patients with lower (17.2 months) vs. higher than median baseline TBR (23.7 months; *p* = 0.08; **a**) at baseline before start of PRRT. Quartile-wise comparison (**b**) of baseline TBR revealed significantly shorter median PFS for patients in the 1st quartile (14.4 months) vs. the 3rd (23.7 months; *p* = 0.03) and 4th quartile (24.1 months; *p* = 0.02) but not vs. the 2nd quartile (22.3 months; *p* = 0.14). No other group differences were observed

**Table 1:** Baseline patient characteristics (*n* = 139)

Age, years		
	Mean (range)	62.1 (27–85)
Sex		
	Male, *n* (%)	78 (56)
	Female, *n* (%)	61 (44)
Primary		
	Pancreas, *n* (%)	47 (34)
	Midgut, *n* (%)	40 (29)
	Unknown, *n* (%)	16 (12)
	Lung/thymus, *n* (%)	12 (9)
	Hindgut, *n* (%)	11 (8)
	Others, *n* (%)	13 (9)
Histopathological grade		
	G1, *n* (%)	35 (25)
	G2, *n* (%)	75 (54)
	G3, *n* (%)	9 (6)
	N/A, *n* (%)	20 (14)
Main metastatic sites		
	Liver, *n* (%)	122 (88)
	Bone, *n* (%)	59 (42)
	Lungs, *n* (%)	5 (4)
TBR		
	TBRmean, mean (range)	22.4 (3.8–169.7)
Prior treatments		
	SSA, *n* (%)	96 (69)
	Chemotherapy, *n* (%)	37 (27)
Baseline Chromogranin A		
	Mean (range) (ng/mL)	2647.2 (19.4–109,424)

*TBR* tumor-to-blood ratio, *SSA* somatostatin analogues, *N/A* not available

### Imaging results

Mean TBR at baseline across all lesions and patients was 22.4. New lesions were observed in 27/139 patients. Increases in TBR were observed in 28/139 patients, 19 of those by > 25%. Decreases in TBR were observed in 84/139 patients, 63 of those by > 25%.

Body weight (*p* = 0.27; *R*^2^ = 0.1) and time post-injection (*p* < 0.001; *R*^2^ =  − 0.36) showed a weak correlation with SUVmean of the blood pool at best.

RECIST 1.1 response at first imaging, within 3–6 months after the last PRRT cycle, was as follows: complete remission 1/139 (1%), partial remission 10/139 (7%), stable disease 83/139 (60%), and progressive disease (PD) in 42/139 (30%) patients. In 3/139 (2%) patients, full diagnostic cross-sectional imaging was missing. Mean TBR for patients with PD was 22.1, for stable disease 22.4, and for partial/complete remission 25.6 (*p* = 0.88). An overview of patient follow-up characteristics is provided in Table 2.

**Table 2:** Follow-up patient characteristics (*n* = 139)

Treatment response		
	PD, *n* (%)	42 (30)
	StD, *n* (%)	83 (60)
	PR, *n* (%)	10 (7)
	CR, *n* (%)	1 (1)
	N/A, *n* (%)	3 (2)
PFS		
	Progression, *n* (%)	107 (77)
	PFS in months, mean (range)	20.1 (2.6–91.1)
OS		
	Deceased, *n* (%)	77 (55)
	OS in months, mean (range)	64.9 (4.3–130.1)

*PD* progressive disease, *PR* partial response, *CR* complete response, *StD* stable disease, *N/A* not available, *PFS* progression-free survival, *OS* overall survival

### Progression-free survival

Mean imaging follow-up until disease progression, or last patient visit without progression, was 22.3 months (range, 2.6–91.1 months). Disease progression was observed in 107/139 (77%) patients.

Median PFS did not differ significantly between patients with baseline TBR below the median (17.0 months) vs. above the median (23.7 months; *p* = 0.06). Quartile-wise comparison of baseline TBR revealed significantly shorter median PFS for patients in the 1st quartile (14.4 months) vs. the 3rd (23.7 months; *p* = 0.03) and 4th quartile (24.1 months; *p* = 0.02) but not vs. the 2nd quartile (22.3 months; *p* = 0.14). Any line of systemic treatment other than SSA was significantly associated with shorter PFS (HR, 1.72 [95%CI, 1.16–2.54]; *p* = 0.006). No other group differences were observed. PFS dependent on baseline TBR is shown in Fig. 1.

After exclusion of patients with PD according to RECIST 1.1 at first follow-up imaging (*n* = 42), the univariate Cox regression model showed a significantly shorter PFS for increasing TBR under PRRT (HR = 2.13 [95%CI = 1.18–3.85]; *p* = 0.01). PFS among patients with RECIST 1.1 complete/partial response vs. stable disease on first follow-up imaging after PRRT was similar (*p* = 0.43).

A further multivariate analysis including tumor grade, as a known prognostic marker [2], confirmed increasing TBR during PRRT as an independent risk factor for early PD (HR = 2.91 [95%CI = 1.54–5.50]; *p* = 0.01). Results of the uni- and multivariate Cox regression model are presented in Table 3; results of a sub-cohort with small-intestine and pancreatic NETs are presented in Supplemental Table 1.

**Table 3:** Prognostic factors for short PFS after PRRT in patients without PD (RECIST) on their first follow-up imaging (*n* = 94)

Parameter	Univariate				Multivariate			
	*p*	HR	95%CI		*p*	HR	95%CI	
			Lower	Upper			Lower	Upper
Increasing TBR	0.02	2.13	1.18	3.85	0.01	2.91	1.54	5.50
Increasing SUV_mean_	0.64	1.24	0.50	3.11				
Bone metastases at baseline	0.05	0.60	0.36	1.00				
Primary	0.96							
Unknown	0.12							
Hindgut*	0.19	3.98	0.51	31.24				
Lung/thymus*	0.65	7.17	0.88	58.14				
Midgut*	0.28	11.12	1.30	95.09				
Pancreas*	0.19	3.85	0.51	28.96				
PPGL*	0.14	4.54	0.61	33.84				
Grade	0.98	1.10	0.63	1.62	0.97	1.01	0.62	1.64
Baseline AP	0.56	1.00	1.00	1.00				
Baseline LDH	0.17	1.00	1.00	1.01				
PR/CR vs. StD	0.43							

^*^Compared with unknown primary

*HR* hazard ratio, *CI* confidence interval, *TBR* tumor-to-blood ratio, *AP* alkaline phosphatase, *LDH* lactate dehydrogenase, *PR* partial response, *CR* complete response, *StD* stable disease

Log-rank test revealed a similar PFS in patients with new lesions (20.0 months) vs. decreasing (27.6 months; *p* = 0.34) or increasing TBR (21.1 months; *p* = 0.89). Patients with increasing TBR showed shorter PFS than those with decreasing TBR (*p* = 0.04). No statistically significant changes were observed for changes in SUVmean (0.41–0.64).

**Fig. 2:**
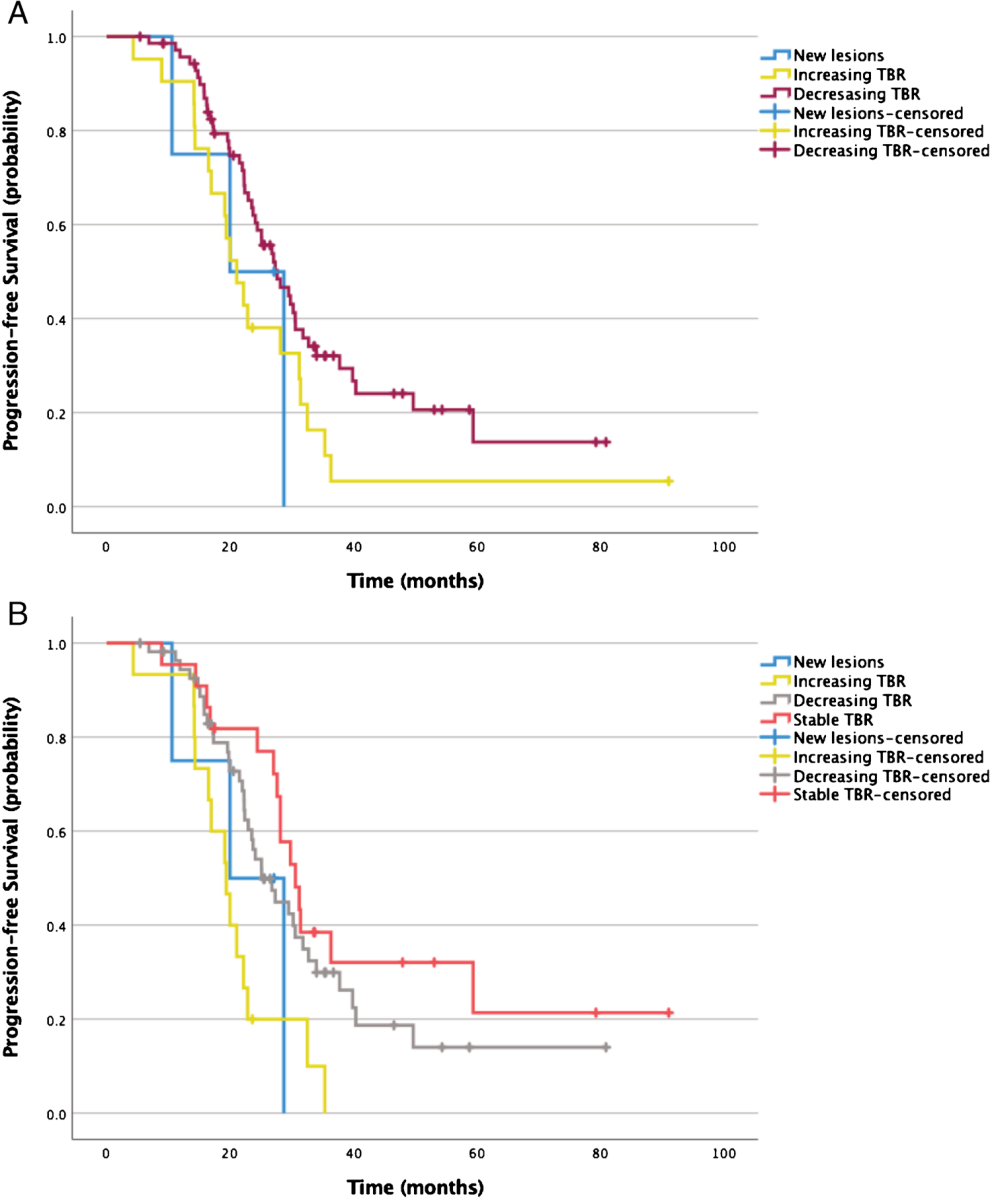
Kaplan–Meier curves plotting PFS of patients with new lesions (*n* = 4) vs. increasing TBR (*n* = 21) vs. decreasing TBR on the first SSTR-PET after PRRT (*n* = 72) (**a**). Median PFS was significantly shorter in patients with increasing (21.1 months) vs. decreasing TBR (27.6 months; *p* = 0.04) but not in patients with new lesions (20.0 months) vs. increasing (*p* = 0.89) TBR or decreasing TBR (*p* = 0.34). With the addition of the category stable TBR (increase/decrease < 25%, **b**), increasing TBR was measured in 15 patients, decreasing TBR in 56 patients, and stable TBR in 22 patients. Median PFS was shorter in patients with increasing TBR (19.4 months) vs. those with stable (30.6 months; *p* = 0.001) or decreasing (25.1 months; *p* < 0.01) TBR. No other statistically significant differences were observed among groups

When introducing the category of stable TBR for increases/decreases < 25%, a significantly shorter PFS was observed in patients with increasing TBR (19.4 months) vs. those with decreasing (25.1 months; *p* < 0.01) or stable (30.6 months; *p* = 0.001) TBR. No statistically significant differences with regard to median OS were observed between patients with new lesions vs. any of the other groups (20.0 months; *p* = 0.13–0.64). Figure 2 shows PFS dependent on changes in TBR.

Median PFS did not differ among patients with new lesions, increasing SUVmean or decreasing SUVmean (*p* = 0.41–0.85).

### Overall survival

Mean follow-up time until death or the last documented date of the patient being alive was 37.9 (range, 4.3–130.1) months. 77/139 (55%) patients died during follow-up.

**Fig. 3:**
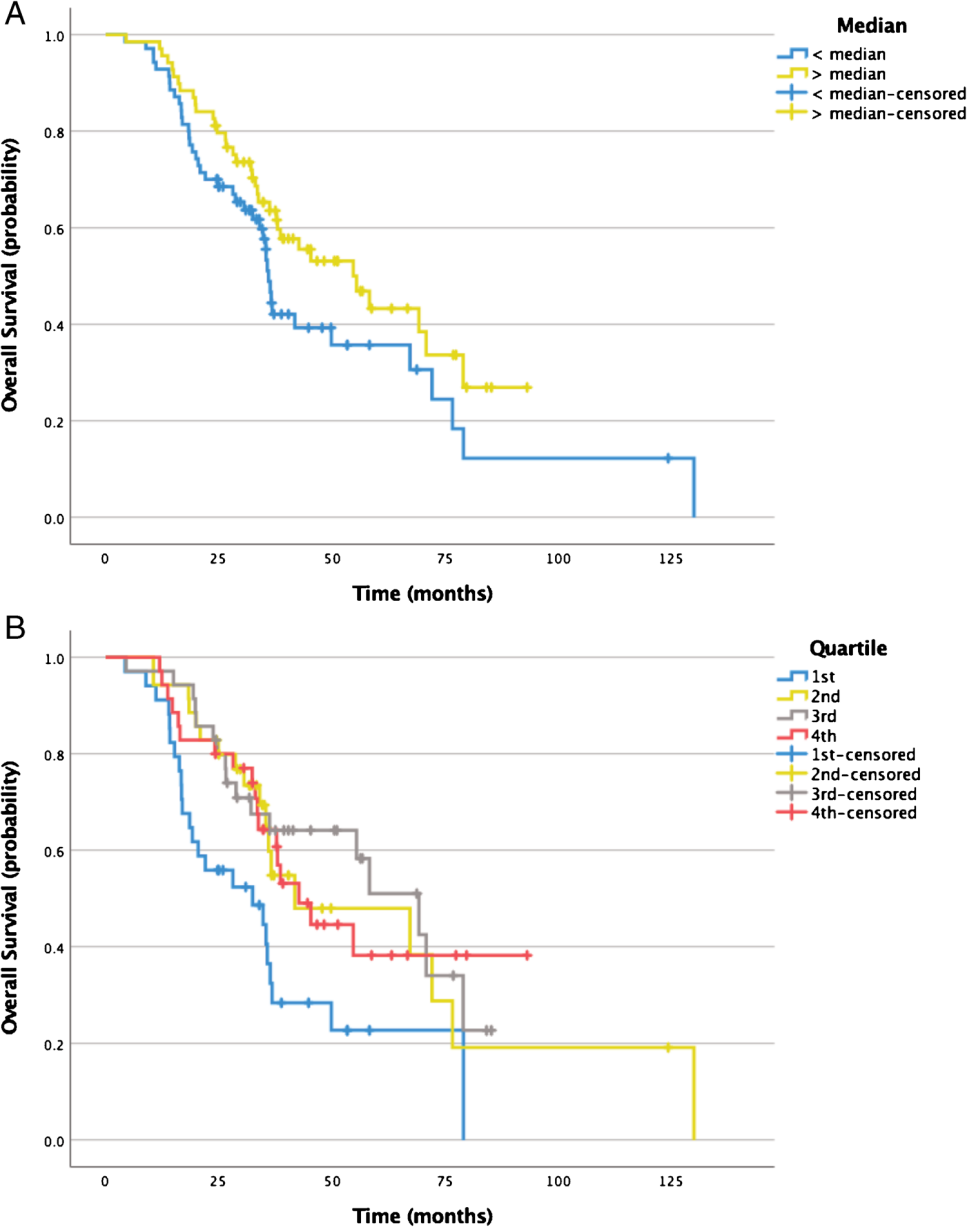
Kaplan–Meier curves plotting OS of patients with lower (*n* = 70) vs. higher (*n* = 69) than median TBR at baseline before PRRT (median OS, 36.0 vs. 55.4 months; *p* = 0.08; **a**). Quartile-wise comparison of baseline TBR (**b**) revealed significantly shorter median OS for patients in the 1st quartile (32.5 months) vs. the 2nd (41.8 months; *p* = 0.03), 3rd (69.2 months; *p* < 0.01), and 4th (42.7 months; *p* = 0.03) quartile. No other group differences were observed

Baseline TBR (*p* = 0.35) was not statistically associated with OS in a univariate Cox regression model. OS was similar in patients with TBR above vs. below median (median OS, 36.0 vs. 55.4 months; *p* = 0.03). Quartile-wise comparison of baseline TBR revealed significantly shorter median OS for patients in the 1st quartile (32.5 months) vs. the 2nd (41.8 months; *p* = 0.03), 3rd (69.2 months; *p* < 0.01), and 4th (42.7 months; *p* = 0.03) quartile. Any line of systemic treatment besides SSA did not bear impact on OS (*p* = 0.10). No other group differences were observed. Comparison of TBR regarding OS is shown in Fig. 3.

A univariate Cox regression model showed that increasing TBR is a significant risk factor for short OS (HR = 1.87 [95%CI = 1.22–2.86]; *p* < 0.01). The absence of progressive disease according to RECIST 1.1 criteria on the first imaging after PRRT was associated with longer OS as well (HR = 0.43 [95%CI = 0.27–0.69; *p* < 0.001). A significant association between OS and changes in SUVmean could not be established (*p* = 0.29).

A multivariate Cox regression model including the parameters tumor grade, PD during PRRT and increases in TBR, confirmed increases in TBR as an independent predictor of short OS (HR = 1.64 [95%CI = 1.03–2.62]; *p* = 0.02). The absence of RECIST 1.1 PD on the first follow-up imaging after PRRT was significantly associated with longer OS (HR = 0.47 [95%CI = 0.28–0.81]; *p* < 0.01). Results of the uni- and multivariate Cox regression model are presented in Table 4; a subcohort analysis on patients with small intestine and pancreas NET is presented in Supplemental Table 2.

**Table 4:** Predictive factors for short OS after PRRT in all patients (*n* = 139)

Parameter	Univariate				Multivariate			
	*p*	HR	95%CI		*p*	HR	95%CI	
			Lower	Upper			Lower	Upper
Increasing TBR	< 0.01	1.87	1.22	2.86	0.04	1.64	1.03	2.62
Increasing SUV_mean_	0.29	1.53	0.70	3.36				
Bone metastases at baseline	0.46	1.19	0.75	1.87				
Primary	0.15							
Unknown								
Hindgut*	0.66	6.65	0.89	50.01				
Lung/thymus*	0.15	4.72	0.58	38.61				
Midgut*	0.11	5.58	0.70	44.66				
Pancreas*	0.23	3.41	0.45	25.69				
PPGL*	0.22	3.52	0.48	25.99				
Grade	0.69	1.10	0.70	1.73	0.68	1.10	0.69	1.75
Baseline AP	0.74	1.00	1.00	1.00				
Baseline LDH	0.19	1.00	1.00	1.01				
No PD at first follow-up	< 0.001	0.43	0.27	0.69	< 0.01	0.47	0.28	0.81

^*^Compared with unknown primary

*HR* hazard ratio, *CI* confidence interval, *TBR* tumor-to-blood ratio, *AP* alkaline phosphatase, *LDH* lactate dehydrogenase, *PD* progressive disease, *PPGL* pheochromocytoma or paraganglioma

Log-rank test showed significantly shorter median OS for patients with new lesions (24.2 months) than for those with decreasing TBR (67.3 months; *p* < 0.001). Median OS was longer for patients with decreasing vs. increasing TBR (34.8 months; *p* = 0.04). Median OS did not differ significantly between patients with new lesions vs. those with increasing TBR (34.8 months; *p* = 0.11).

There were statistically significant differences with regard to median OS in patients with new lesions (24.2 months) vs. those with decreasing (28.2 months; *p* < 0.001) or increasing (24.5 months; *p* < 0.01) SUVmean. However, median OS did not differ significantly between patients with increasing vs. decreasing SUVmean (*p* = 0.55).

When classifying patients with minor (< 25%) changes in TBR as stable disease, significantly shorter median OS was observed for patients with new lesions (24.2 months) than for those with stable (54.7 months; *p* < 0.01) or decreasing TBR (69.2 months; *p* < 0.001).

**Fig. 4:**
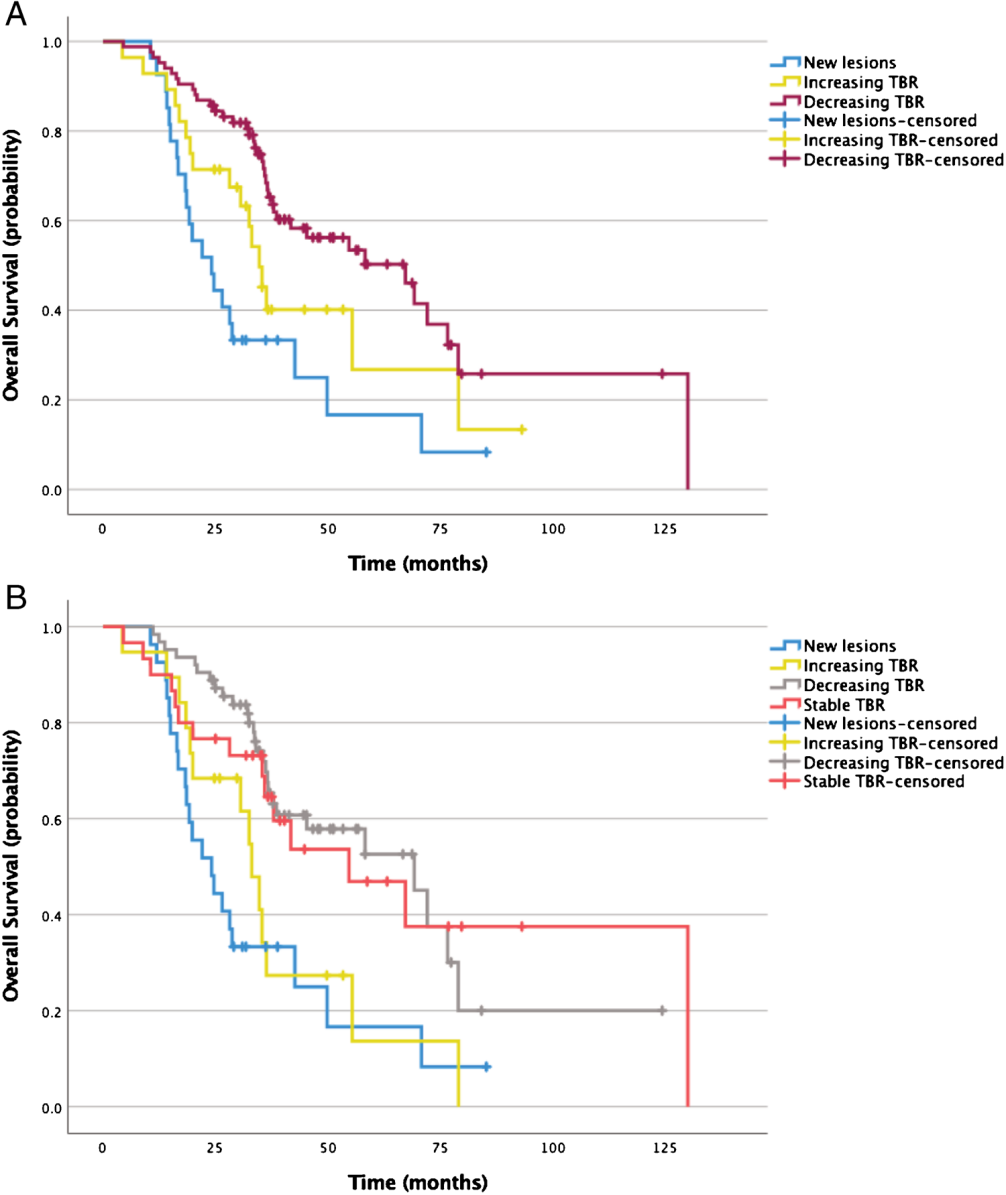
Kaplan–Meier curves plotting OS of patients with new lesions (*n* = 27) vs. increasing TBR (*n* = 28) vs. decreasing TBR (*n* = 84, **a**) on the first SSTR-PET after PRRT. Median OS was significantly shorter in patients with new lesions (24.2 months) vs. decreasing TBR (67.3 months; *p* < 0.001) and in patients with increasing TBR (34.8 months) vs. decreasing TBR (*p* = 0.04). With the addition of the category stable TBR (increase/decrease < 25%, **b**), increasing TBR was measured in 19 patients, decreasing TBR in 63 patients, and stable TBR in 30 patients. OS was different among patients with new lesions (24.2 months) vs. stable TBR (54.7 months; *p* < 0.01) and vs. decreasing TBR (69.2 months; *p* < 0.001). OS was also shorter in patients with increasing TBR (33.1 months) vs. decreasing TBR (*p* < 0.01) and patients with increasing vs. stable TBR (*p* = 0.04). No statistically significant differences were found between patients with new lesions vs. increasing TBR (*p* = 0.40), patients with stable vs. decreasing TBR (*p* = 0.82)

The mean OS was shorter in patients with increasing TBR (33.1 months) than in those with decreasing (*p* < 0.01) or stable TBR (*p* = 0.04). OS did not differ significantly in patients with stable vs. decreasing TBR (*p* = 0.82). Comparison of OS based on changes in TBR is shown in Fig. 4.

No statistically significant association was observed for median OS and increasing vs. stable (*p* = 0.35), increasing vs. decreasing (*p* = 0.29), and stable vs. decreasing SUVmean (*p* = 0.63).

## Discussion

This study is the first to show that baseline to follow-up changes in TBR are a robust tool for the assessment of treatment response in NET patients undergoing PRRT.

Decreases in TBR were significantly associated with longer PFS and OS, whereas increases were associated with a shorter PFS and OS. Even when accounting for RECIST 1.1 response and known risk factors for grim outcome, increasing TBR under treatment was still a significant risk factor for short OS and PFS, further underlining the independent value of TBR as a metric for response assessment. Patients with increasing TBR were at higher risk for early progression and death and may therefore benefit from closer surveillance with shorter follow-up intervals and/or earlier interventions. A 25% threshold further improved the prognostic performance, presumably by aiding differentiation of unspecific fluctuations from actual increases in SSTR expression. Of note, in our cohort, the impact of an increase of TBR by > 25% on OS and PFS was comparable to the occurrence of new lesions.

Monitoring of changes in TBR is of particular relevance in the majority of patients without PD after PRRT, where in our cohort, it was the only parameter significantly associated with OS. In future prospective trials, the optimal follow-up timepoint for TBR also during PRRT needs to be explored. In line with prior studies, we confirmed primary in the lung/thymus [2], high baseline LDH levels [19], and progression during PRRT [2] as negative prognostic markers. In contrast, changes in SUVmean were not associated with patient outcome. These superior stratification capabilities of TBR over SUVmean could not be sufficiently explained by potential confounders, such as body weight or time post-injection.

Unexpectedly, tumor grade was not predictive of PFS and OS after PRRT. As only a fraction of G3-NETs is SSTR positive, the patients included in this study are likely not representative of G3-NETs in general. Furthermore, the number of G3 patients was relatively low, and they were more likely to receive PRRT as first-line treatment. A further explanation may be constituted by the observed intertumoral heterogeneity in NETs, i.e., the tumor grade determined in the primary tissue may not be reflective of the tumor grade of other metastatic sites within the same patient [20].

This study is also the first to evaluate baseline TBR as a prognostic metric for treatment response. This is of particular importance, since enrolment in many well-studied historical cohorts, such as in the NETTER-1 study (1) or in the ERASMUS study (2), was based on SRS, which has gradually been replaced by SSTR-PET, due to its improved diagnostic performance. Since the uptake intensity between SRS and SSTR-PET may vary, especially in superficial lesions close to the gamma camera detectors and in small to intermediate sized lesions [6], well-established PET parameters that predict treatment response are scarce.

Following prior studies that show a high correlation between TBR and somatostatin receptor expression [13] and the assumption that high SSTR expression in the baseline examination increases the likelihood of treatment response due to higher absorbed tumor doses, we evaluated TBR as a predictor of treatment response. We could show significantly shorter OS and PFS for patients with a TBR in the first quartile. The high risk of treatment failure in this patient cohort may warrant discussion of other treatment modalities. The role of baseline TBR for patient selection needs to be further prospectively explored.

Strengths of this study are the large number of patients, the long follow-up leading to a high number of registered PFS and OS events, and that the imaging analysis was performed by two independent and blinded readers. Also, the measured tumors represented a spectrum of lesions, from low-uptake to high-uptake lesions, further emphasizing the robustness of the assessed metrics.

Limitations are the retrospective single-center design, as well as the lack of a validation cohort. Although, up to nine tumor lesions per patient were chosen based on their level uptake in relation to normal organs and tissues (low, intermediate, and high), other factors such as their suitability for delineation and reader’s subjective assessment of their representativeness may potentially have biased the results. Rapid emergence of semi-automatic segmentation tools may facilitate assessment of all tumor lesions in each patient, to improve interobserver agreement and eliminate lesion selection bias. Additional caveats are constituted by (i) low administered activities potentially affecting lesion quantification by increasing image noise, (ii) varying uptake times (although these may be partly offset by use of TBR), and (iii) the low number of patients with G3-NETs limiting conclusions on this particular patient cohort.

## Conclusion

Assessment of baseline to follow-up changes in TBR offers added value to standalone morphological imaging for response evaluation in NET patients undergoing PRRT. Baseline TBR may aid the identification of patients least likely to benefit from PRRT. Further studies are warranted to confirm these results.

### Supplementary Information

Below is the link to the electronic supplementary material.

Supplementary file1 (PNG 312 KB) Supplemental Figure 1. Patient selection process.

Supplementary file2 (DOCX 41 KB)
