# Supplementary figures and images for: Changes in tumor-to-blood ratio as a prognostic marker for progression-free survival and overall survival in neuroendocrine tumor patients undergoing PRRT
Source: Eur J Nucl Med Mol Imaging. 2023 Nov 10;51(3):841–51. doi: 10.1007/s00259-023-06502-y (PMC10796732; doi:10.1007/s00259-023-06502-y)

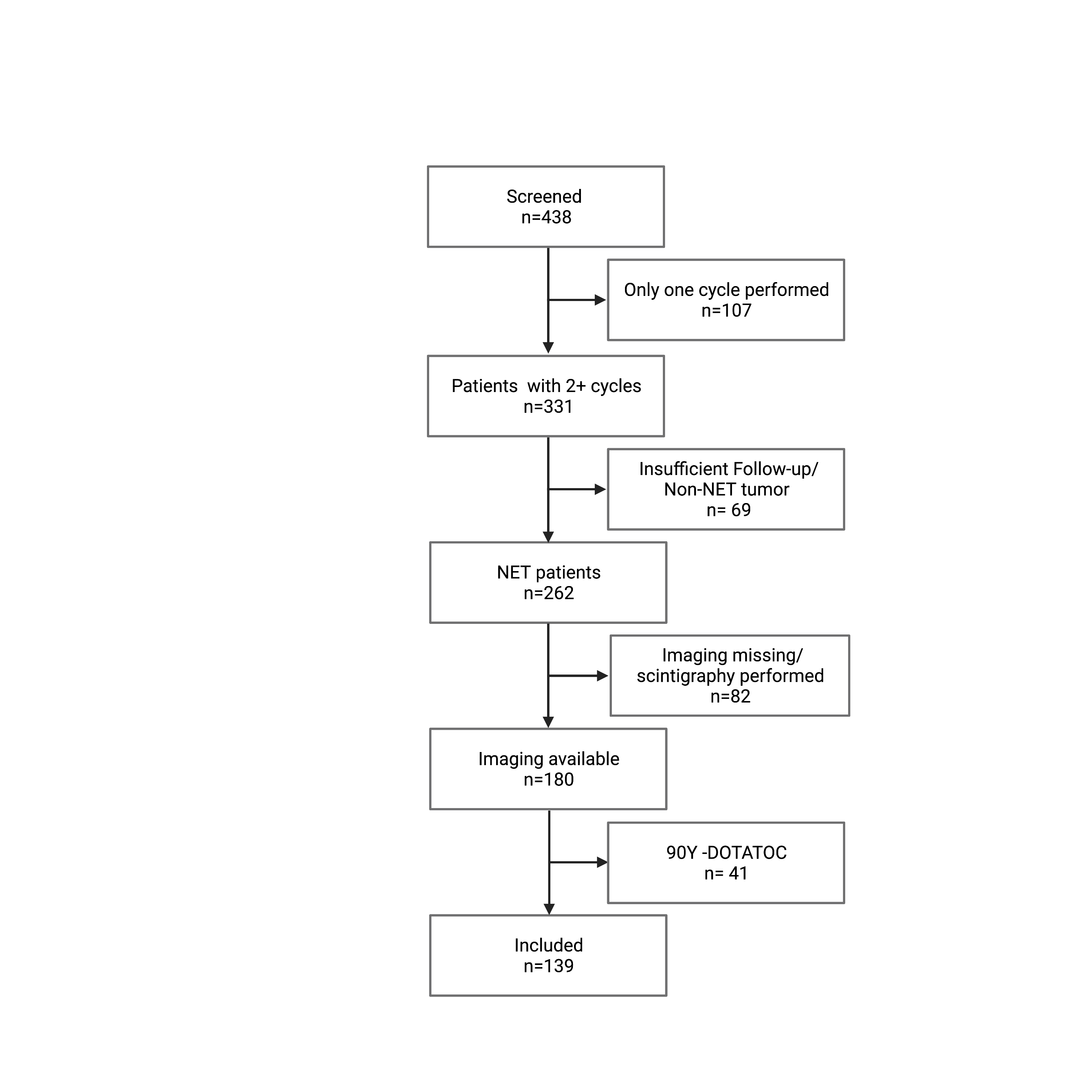

Supplement: Supplementary file 1 — Supplementary file1 (PNG 312 KB) Supplemental Figure 1. Patient selection process. [file 259_2023_6502_MOESM1_ESM.png]
