# Supplementary material for: Changes in tumor-to-blood ratio as a prognostic marker for progression-free survival and overall survival in neuroendocrine tumor patients undergoing PRRT
Source: Eur J Nucl Med Mol Imaging. 2023 Nov 10;51(3):841–51. doi: 10.1007/s00259-023-06502-y (PMC10796732; doi:10.1007/s00259-023-06502-y)
Supplement: Supplementary file 2 — Supplementary file2 (DOCX 41 KB) [file 259_2023_6502_MOESM2_ESM.docx]

**Supplemental Table 1.** Prognostic factors for short PFS after PRRT in NET patients with of small-intestinal and pancreatic origin without PD (RECIST) on their first follow-up imaging (n=65). HR=Hazard Ratio; CI=confidence interval; TBR=Tumor-to-Blood-ratio; AP=alkaline phosphatase; LDH= lactate dehydrogenase; PR= partial response; CR=complete response; StD=stable disease.

| Parameter | | Univariate | | | | Multivariate | | | |
| --- | --- | --- | --- | --- | --- | --- | --- | --- | --- |
|  |  | p= | HR | 95%CI | | p= | HR | 95% CI | |
|  |  |  |  | Lower | Upper |  |  | Lower | Upper |
| Increasing TBR | | <0.001 | 3.76 | 1.83 | 7.69 | 0.01 | 3.60 | 1.71 | 7.60 |
| Increasing SUV_mean_ | | 0.39 | 1.66 | 0.52 | 5.30 |  |  |  |  |
| Bone metastases at baseline | | 0.01 | 0.34 | 0.15 | 0.77 | 0.02 | 0.32 | 0.13 | 0.80 |
| Primary | |  |  |  |  |  |  |  |  |
|  | Pancreas* | 0.57 | 0.84 | 0.46 | 1.53 |  |  |  |  |
| Grade | | 0.81 | 1.07 | 0.65 | 1.78 | 0.40 | 1.27 | 0.73 | 2.23 |
| Baseline AP | | 0.31 | 1.00 | 1.00 | 1.00 |  |  |  |  |
| Baseline LDH | | 0.82 | 1.00 | 0.99 | 1.01 |  |  |  |  |
| PR/CR vs. StD | | 0.15 | 0.57 | 0.27 | 1.22 |  |  |  |  |

*Compared to small-intestine NET

**Supplemental Table 2** Predictive factors for short OS after PRRT in all patients with small-intestine and pancreas NET (n=187). HR=Hazard Ratio; CI=confidence interval; TBR=Tumor-to-Blood-ratio; AP=alkaline phosphatase; LDH= lactate dehydrogenase; PD=progressive disease.

| Parameter | | Univariate | | | | Multivariate | | | |
| --- | --- | --- | --- | --- | --- | --- | --- | --- | --- |
|  |  | p= | HR | 95% CI | | p= | HR | 95% CI | |
|  |  |  |  | Lower | Upper |  |  | Lower | Upper |
| Increasing TBR | | <0.01 | 2.57 | 1.33 | 4.96 | 0.06 | 2.10 | 0.97 | 4.53 |
| Increasing SUV_mean_ | | 0.26 | 1.71 | 0.67 | 4.39 |  |  |  |  |
| Bone metastases at baseline | | 0.68 | 0.87 | 0.45 | 1.68 |  |  |  |  |
| Primary | |  |  |  |  |  |  |  |  |
|  | Pancreas* | 0.89 | 0.96 | 0.52 | 1.78 |  |  |  |  |
| Grade | | 0.46 | 0.80 | 0.44 | 1.45 | 0.68 | 1.10 | 0.69 | 1.75 |
| Baseline AP | | 0.89 | 1.00 | 1.00 | 1.00 |  |  |  |  |
| Baseline LDH | | 0.44 | 1.00 | 1.00 | 1.01 |  |  |  |  |
| No PD at first follow-up | | <0.01 | 0.43 | 0.23 | 0.80 | 0.03 | 0.47 | 0.24 | 0.94 |

*Compared with small-intestine NET
